# Comparison of auditory brainstem response and electrocochleography to assess the coupling efficiency of active middle ear implants

**DOI:** 10.3389/fneur.2023.1231403

**Published:** 2023-09-06

**Authors:** Tom Gawliczek, Georgios Mantokoudis, Lukas Anschuetz, Marco D. Caversaccio, Stefan Weder

**Affiliations:** ^1^Department of ENT, Head and Neck Surgery, Bern University Hospital, University of Bern, Bern, Switzerland; ^2^Hearing Research Laboratory, ARTORG Center for Biomedical Engineering Research, University of Bern, Bern, Switzerland

**Keywords:** active middle ear implant, coupling efficiency, objective measures, electrocochleography, auditory brainstem response, Vibrant Soundbridge

## Abstract

**Aim:**

This study aimed to compare the effectiveness of auditory brainstem response (ABR) and extracochlear electrocochleography (ECochG) in objectively evaluating the coupling efficiency of floating mass transducer (FMT) placement during active middle ear implant (AMEI) surgery.

**Methods:**

We enrolled 15 patients (mean age 58.5 ± 19.4 years) with mixed hearing loss who underwent AMEI implantation (seven ossicular chain and eight round window couplings). Before the surgical procedure, an audiogram was performed. We utilized a clinical measurement system to stimulate and record intraoperative ABR and ECochG recordings. The coupling efficiency of the VSB was evaluated through ECochG and ABR threshold measurements. Postoperatively, we conducted an audiogram and a vibrogram.

**Results:**

In all 15 patients, ABR threshold testing successfully determined intraoperative coupling efficiency, while ECochG was successful in only eight patients. In our cohort, ABR measurements were more practical, consistent, and robust than ECochG measurements. Coupling efficiency, calculated as the difference between vibrogram thresholds and postoperative bone conduction thresholds, was found to be more accurately predicted by ABR measurements (*p* = 0.016, *R*^2^ = 0.37) than ECochG measurements (*p* = 0.761, *R*^2^ = 0.02). We also found a non-significant trend toward better results with ossicular chain coupling compared to round window coupling.

**Conclusion:**

Our findings suggest that ABR measurements are more practical, robust, and consistent than ECochG measurements for determining coupling efficiency during FMT placement surgery. The use of ABR measurements can help to identify the optimal FMT placement, especially with round window coupling. Finally, we offer normative data for both techniques, which can aid other clinical centers in using intraoperative monitoring for AMEI placement.

## 1. Introduction

Active middle ear implants (AMEIs) are medical devices intended for the treatment of hearing loss by direct stimulation of the middle ear structures. The Vibrant Soundbridge (MED-EL, Austria) is currently the most commonly used implant (1, 2). AMEIs are employed to provide amplification in individuals with hearing loss who are unable to use conventional hearing aids due to issues with their outer or middle ear (3). These include chronic infections of the outer or middle ear, atresia or stenosis of the ear canal, or problems with feedback when using conventional hearing aids. They may be recommended for patients with sensorineural, conductive, or mixed hearing loss (4).

The external component of the AMEI is a sound processor that transmits the auditory signal digitally to the implant. The implantable part comprises a coil, a magnet, a demodulator, and a floating mass transducer (FMT). One of the significant benefits of the MED-EL Soundbridge is its adaptability concerning surgical placement. Depending on the individual's anatomy and hearing loss characteristics, the FMT can be attached to the ossicular chain (i.e., the incus or stapes), the round window, or the oval window. However, suboptimal placement of the FMT can negatively affect sound amplification and patient satisfaction (4, 5). One major reason for poor coupling is the large number of degrees of freedom (for example, on the round window, the FMT can be placed differently into the niche (6, 7). There is a high number of reported subobtimal FMT placements with associated revisions surgeries (6, 8–10). Any solution offering to the surgeon an intraoperative objective evaluation of the coupling efficiency is therefore crucial.

Neurophysiological recordings, such as extracochlear electrocochleography (ECochG) and auditory brainstem response (ABR) measurements, can aid in identifying the optimal FMT placement. Prior research has demonstrated that ECochG recordings can enhance surgical techniques for round window placement (6), while ABR potentials have been utilized to evaluate FMT placement at different anatomical locations (9–11).

Despite these findings, a direct comparison between the two recording techniques, including signal analysis, is lacking. Therefore, our study aimed to address this gap and determine the feasibility, surgical aspects, and coupling efficiency of the two methods. We considered the technical setup, audiological assessment, and coupling modalities to determine whether ECochG and ABR are equivalent in terms of providing accurate and reliable information for optimizing FMT placement.

## 2. Materials and methods

### 2.1. Study design and demographics

Our exploratory study was executed in compliance with the principles outlined in the Declaration of Helsinki and the regulations established by the local ethics commission (BASEC-ID no. 2019-00555). Written informed consent was obtained from all participants. The study enrolled 15 patients who underwent implantation of a Vibrant Soundbridge VORP503 (MED-EL, Austria) between May 2021 and March 2023.

### 2.2. Pre-operative and postoperative assessments

Before the surgical procedure, an audiogram was performed on all participants within a sound attenuated acoustic chamber using a calibrated device (Interacoustics, Denmark). This evaluation included the assessments of air conduction (AC) and bone conduction (BC) thresholds in dB HL. Pure tone average (PTA) was calculated from measurements at 1,000, 2,000, and 4,000 Hz for the implanted side (8, 12). Four weeks after the implantation, we assessed BC and vibrogram thresholds with implant (latter in dB HL eq.). The demographic and audiological evaluations of the participants are presented in Table 1.

**Table 1:** Characteristics of the 15 subjects.

**No**.	**Sex**	**Side**	**Type**	**Coupler**	**Disease leading to hearing loss**	**Number of previous ear surgeries**	**Reasons for Soundbridge surgery**	**Surgery**	**Preoperative**			**Intraoperative**		**Post-operative**
									**(dB HL)**			**(dB nHL)**		**(dB HL eq.)**
									**BC**	**AC**	**ABG**	**ABR**	**ECochG**	**Vibrogram**
1	m	Left	Imp	RW	Cholesteatoma	3	Infected radical cavity,increasing mixed hearing loss	Subtotal petresectomy with blind sack closure, RW coupling	47 (20–65)	98 (80–110)	51	60	Nm	45 (40-50)
2	f	Left	Imp	SH	Cholesteatoma	2	Increasing mixed hearing loss; extrusion of Partial Ossicular Chain Replacement (PROP)	Combined transcanal-transmastoid placement of SH stapes coupler	33 (15–45)	85 (75–105)	52	55	Nm	48 (35–70)
3	m	Left	Rev	RW	Complex petrous bone fracture with combined hearing loss	2	FMT dislocation	Transcanal revision of FMT placement RW niche	60 (55–65)	92 (90–95)	32	80	Nm	63 (55–75)
4	m	Left	Rev	RW	Chronic otitis media	4	FMT dislocation	Transcanal revision of FMT placement RW niche	28 (15–35)	85 (75–105)	57	60	50	42 (30–50)
5	m	Right	Imp	RW	Cholesteatoma	2	Infected radical cavity	Subtotal petresectomy with blind sack closure, RW coupling	38 (30–50)	62 (55–70)	24	60	70	67 (60–80)
6	f	Right	Imp	SH	Mucoepidermoid carcinoma of the parotid gland	0	Post-irradiation osteoradionecrosis of the petrous bone	Subtotal petresectomy with blind sack closure, SH stapes coupling	53 (45–60)	67 (60–75)	14	60	Nm	67 (60–75)
7	m	Right	Imp	SH	Nasopharyngeal carcinoma	0	Post-irradiation osteoradionecrosis of the petrous bone	Subtotal petresectomy with blind sack closure, SH stapes coupling	40 (10–55)	67 (30–100)	27	80	80	62 (45–75)
8	m	Left	Imp	SH	Cholesteatoma	5	Recurring Cholesteatoma and increasing mixed hearing loss	Subtotal petresectomy with blind sack closure, SH stapes coupling	40 (25–50)	98 (85–115)	58	50	70	53 (35–65)
9	m	Left	Imp	RW	Tympanosclerosis	4	Increasing mixed hearing loss	Transmastoid placement of RW coupling	40 (25–50)	80 (70–95)	40	70	80	60 (40–70)
10	m	Left	Imp	RW	Explosion trauma and subsequent cholesteatoma	1	Increasing mixed hearing loss	Revision radical cavity, RW coupling	20 (10–30)	78 (75–80)	58	40	50	30 (20–40)
11	f	Left	Rev	SH	Inability to use conventional hearing aids	1	Increasing mixed hearing loss	Combined transcanal-transmastoid placement of SH stapes coupler	67 (55–75)	82 (75–95)	15	75	Nm	50 (45–60)
12	m	Left	Imp	RW	Tympanosclerosis	0	High degree of mixed hearing loss	Transmastoid placement of RW coupling	33 (15–45)	97 (75–115)	64	80	Nm	87 (80–95)
13	m	Right	Imp	SH	Nasopharyngeal carcinoma	1	Post-irradiation osteoradionecrosis of the petrous bone	Subtotal petresectomy with blind sack closure, SH stapes coupling	35 (10–60)	53 (35–90)	18	55	70	52 (35–80)
14	f	Right	Imp	RW	Tympanosclerosis	1	High degree of mixed hearing loss	Transmastoid placement of RW coupling	42 (35–50)	77 (75–80)	35	65	Nm	58 (50–65)
15	m	Left	Imp	INC	Sudden Sensineural Hearing Loss	0	No success with conventional hearing aids	Transmasoid placement of shot incus coupling	55 (50–60)	62 (55–65)	7	70	70	78 (75–85)

The AC, BC, and vibrogram thresholds show the average values across three frequencies (1, 2, and 4 kHz) with their minimum and maximum values. Improvement the coupling modalities consist of round window coupler (RW) and ossicular chain coupling, which involve the use of Stapes coupler (SH) and Incus coupler (INC). The types of surgery were referred to as implantation (Imp) and revision surgery (Rev). Preoperative air conduction (AC) and bone conduction (BC) thresholds, intraoperative thresholds (ABR and ECochG), air-bone gap (ABG) and postoperative *in situ* thresholds with implant (Vibrogram).

### 2.3. Intraoperative measurement setup

**Figure 1:**
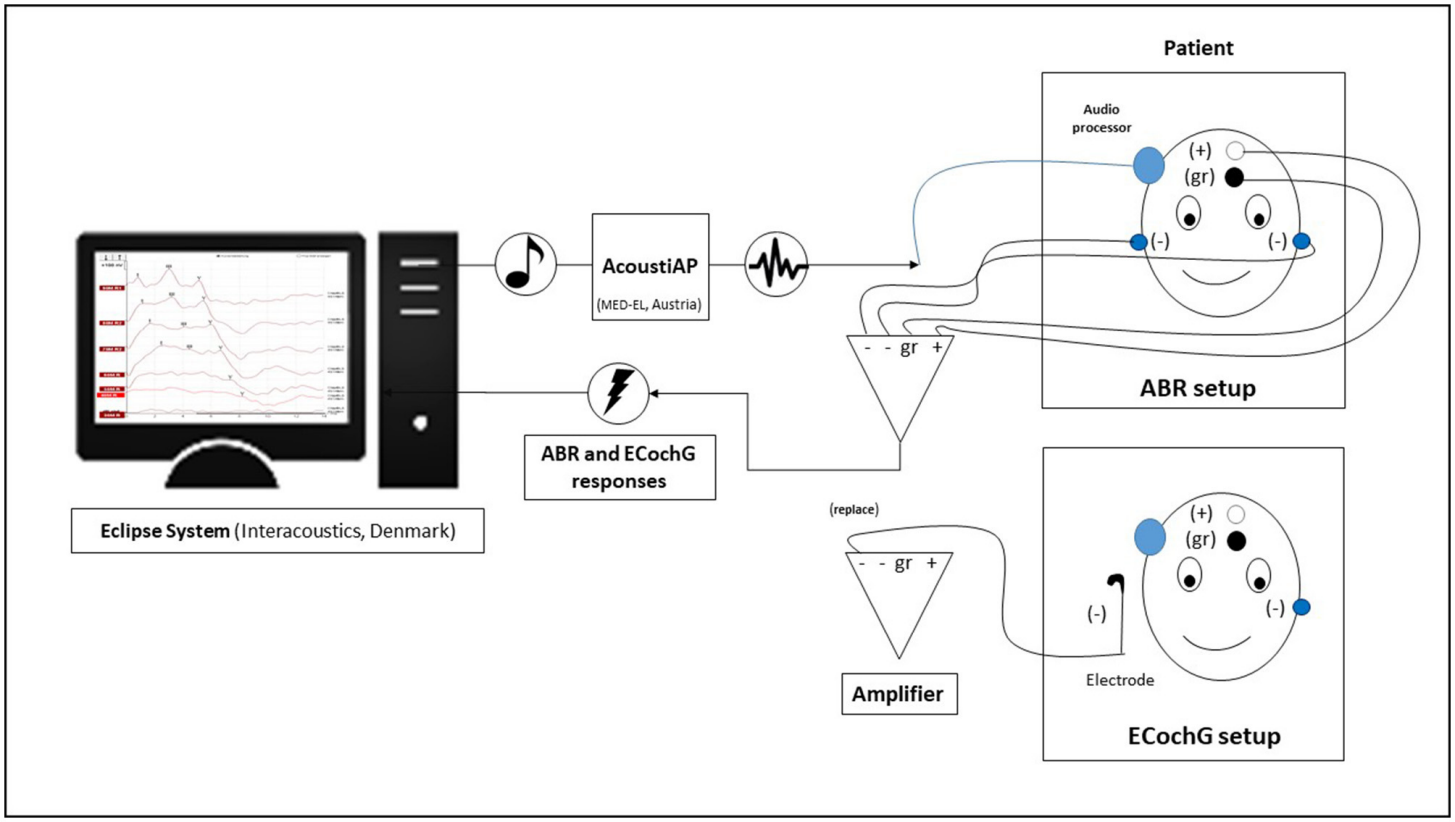
The diagram shows the intraoperative setup using the Eclipse system. The electrodes were taped differently for auditory brainstem response (ABR) and electrocochleography (ECochG) measurements.

We utilized a clinical measurement system to stimulate and record all intraoperative electrophysiological recordings (Eclipse, EP version 4.6, Interacoustics A/S, Denmark). To facilitate stimulation, we connected a Vibrant Soundbridge audio processor to the implant by wrapping it in a sterile bag and attaching it to the coil of the implant, which had already been positioned and fixed in a subperiosteal pocket in its final location. At the opposite end, we connected the audio processor to an AcousticAP device (MED-EL, Innsbruck, Austria), which enabled us to connect the measurement system to the Eclipse system. The AcousticAP with audio processor generated a calibrated signal referenced to the in-ear headphones (IP30 insert phone speaker, 50 ohm) of the Eclipse system. To maintain consistency, we utilized the output/voltage intensity levels calibration as provided by the manufacturer. Figure 1 shows a schematic of the measurement setup.

For recording purposes, we positioned adhesive recording electrodes on the head, with a distance of approximately 1.5 cm between the “+” electrode and the ground “gr” electrode. The reference electrodes (“−”) were positioned on the ipsilateral neck and contralateral mastoid, respectively. Prior to the start of the measurements, we ensured that all adhesive electrodes had an impedance lower than 3 kOhm. For measurements, we initially set a noise rejection level of 80 μV. In situations with significant background noise, a decision was made to increase the suppression level to 320 V in a specific case. This adjustment also resulted in an increase in the minimum number of sweeps to 2,500. The objective was to attain a residual noise level of 60–40 nV or lower, adhering to the manufacturer's system recommendation, in order to precisely assess thresholds.

### 2.4. Intraoperative data collection

**Figure 2:**
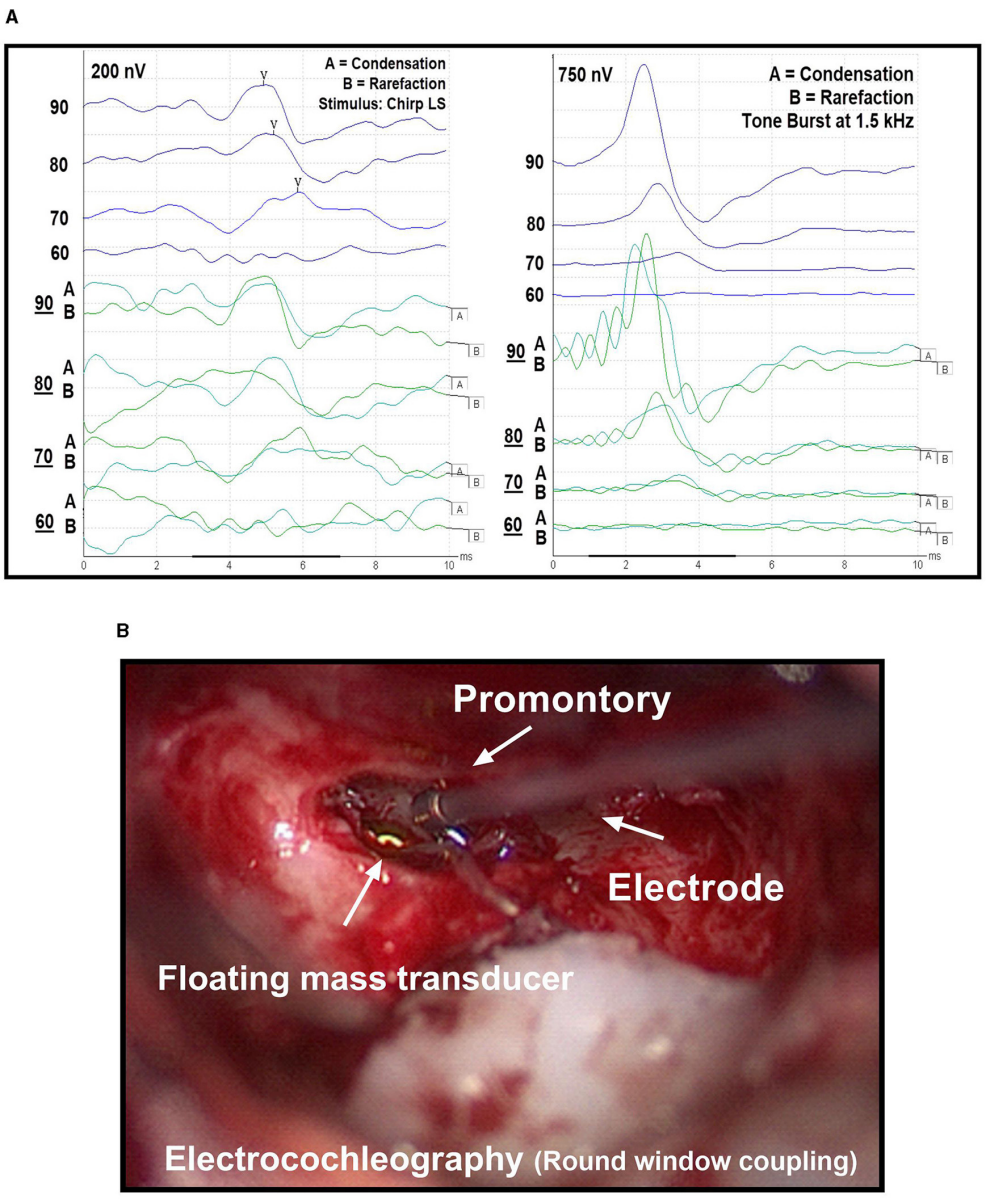
**(A)** Threshold measurement using auditory brainstem response (ABR, left) and electrocochleography (ECochG, right). In both methods, a 90 dB stimulus is gradually reduced in 10 dB steps until no response is visible. Rarefaction and condensation levels are represented by **(A, B)** curves, respectively. The average of the summation of both curves results in the overall curve. **(B)** Extracochlear electrocochleography (ECochG) measurement using a commercially available electrode, which is held on the promontory during recordings.

We conducted intraoperative electrophysiological measurements immediately after placement of the FMT. For stimulation during ABR measurements, we used a broadband LS CE-Chirp with a stimulation frequency of 49.1 Hz at alternating polarities (condensation and rarefaction). We chose this approach because previous research has shown that it increases the amplitude of the signal (13). After the definitive placement of the FMT, the ABR measurement procedure started with a stimulus intensity of 90 dB, followed by reduction in steps of 10 dB until no signal was visible (Figure 2A). In four cases, an additional measurement at the threshold level using a 5 dB step interval could be conducted due to time constraints during the surgical procedure (Table 1). The electrophysiological threshold was confirmed with a second measurement. If no electrophysiological response was observed at 90 dB or the threshold was high, the FMT was re-positioned and the ABR measurements were repeated until the clearest possible signal was obtained. If multiple ABR measurements were performed, only those values with the final FMT position were used in subsequent data analysis.

In a next step, we performed the extracochlear ECochG measurements (Figure 2B). The surgeon placed a sterile electrode (PromStim, MED-EL, Austria) on the promontory, connected to the ipsilateral channel of the preamplifier of the recording system. Impedance values were monitored and the electrode repositioned as required until a value of less than 20 kOhm was achieved. For stimulation, sinusoidal tone bursts at a frequency of 1.5 kHz (with a Blackman function) were used (8). As described above, ECochG measurements started at 90 dB and were lowered step-wise by 10 dB. However, the positioning of the FMT was not changed, as the final placement had previously been determined using the ABR method.

For both electrophysiological recordings, signal analysis was performed visually by two experts. In case of no consensus among experts, the higher threshold value was considered. Only signals with a response in both the condensation and rarefaction measurements were considered. During the ABR recordings, they paid attention to the occurrence and course of the wave V. For the ECcochG responses, they looked at the summation potential (summation of the response to the condensation and rarefaction stimulus).

### 2.5. Data analysis

**Figure 3:**
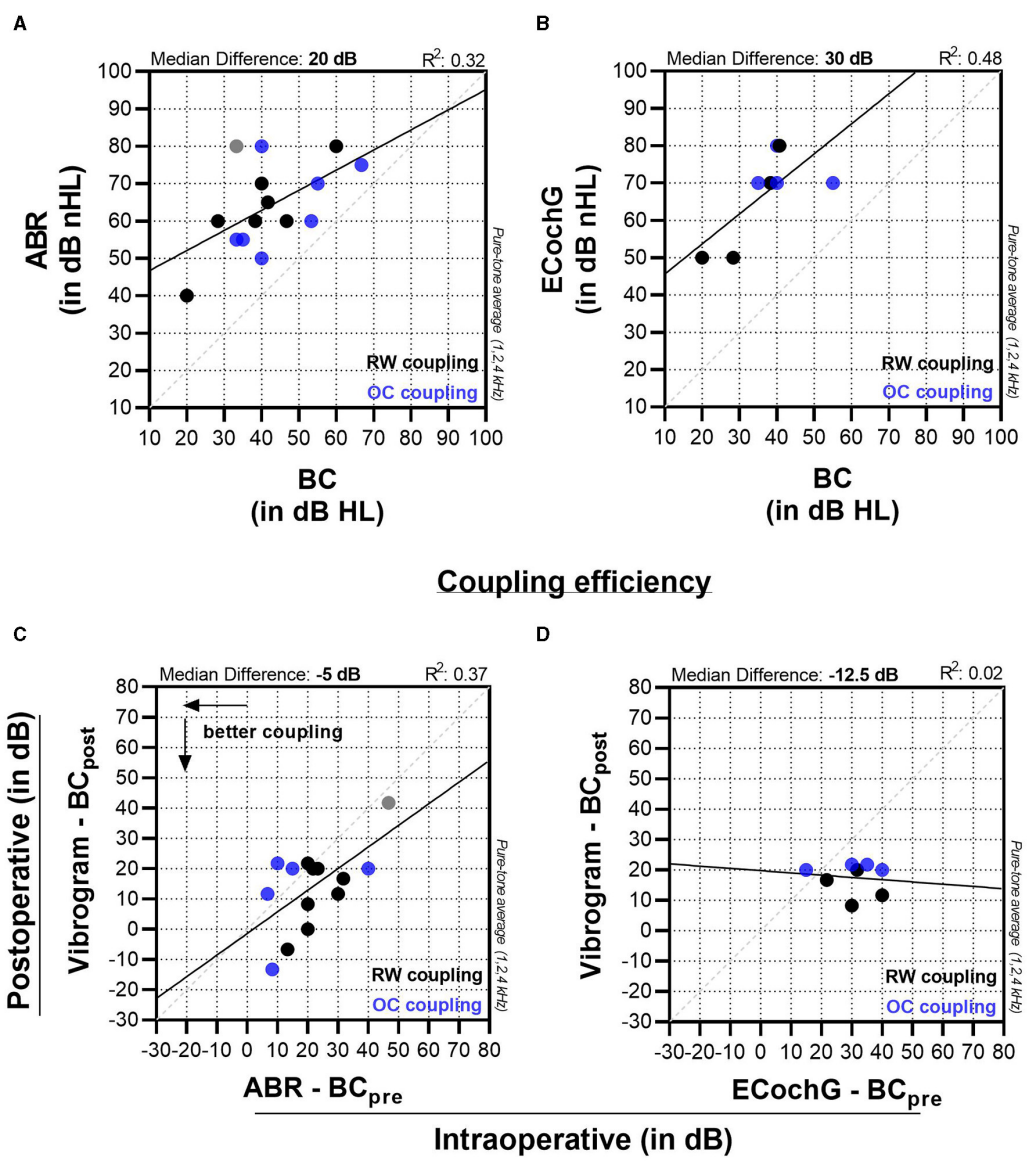
Panels **(A, B)** display the comparison of intraoperative ABR and ECochG measurements to preoperative bone conduction (BC) thresholds. Graphs **(C, D)** compare the coupling efficiency (vibrogram thresholds—postoperative BC thresholds) to the intraoperatively measured threshold (intraoperative ABR—preoperative BC threshold).

We used GraphPad Prism 9.3.1 software (GraphPad Software, USA) for statistical analysis and data visualization. First, we assessed our data using Pearson correlation. Thereby, we compared intraoperative measurements (ABR and ECochG) to preoperative BC thresholds (Figures 3A, B). Figures 3C, D illustrate the relative coupling efficiency for both ABR and ECochG measurements. To create these graphs, we subtracted the postoperative BC threshold from the postoperative vibrogram threshold and plotted the resulting value against the difference between the intraoperative threshold and the preoperative bone conduction threshold. Finally, we compared subjects with ossicular chain (OC) and round window (RW) coupling modalities in respect to coupling efficiency. For this analysis a Mann-Whitney test was performed with a statistical significance level of 0.05.

## 3. Results

### 3.1. Patient demographics

We included 15 patients who underwent implant surgery (Vibrant Soundbridge VORP 503, MED-EL, Austria), including three revision cases (Table 1). Eleven males and four females were on average 58.5 years old (range 39–79 years). The FMT was connected in six cases to the OC (six stapes head couplers and one incus coupler) and eight times to the RW. The pure tone average hearing thresholds after surgery (BC 43.2 dB HL SD ± 13.7; AC 85.3 dB HL, SD ± 12.6) were almost identical to the preoperative BC threshold (42.1 dB HL, SD ± 12.4) and somewhat lower for the AC threshold (78.8 dB HL, SD ± 14.3).

### 3.2. Electrophysiological recordings

We were able to successfully measure an ABR response in all 15 cases. For the ECochG measurements, this was only the case in 8 subjects. In two cases, significant impedance fluctuations were observed. One possible reason is the entry of blood traces into the surgical site, which causes impedance changes. At the same time, stable electrode positioning is made more difficult. In three cases, the signal-to-noise ratio was too low to record meaningful measurements. In one of these cases, the ECochG traces were affected by a second synchronous signal, possibly due to the patient's pacemaker. Finally, in two patients, threshold measurements were started but could not be completed due to recurrent signal loss. After several attempts, the measurements were stopped in order not to prolong the anesthesia time unnecessarily.

For ABR measurements in our cohort, the experts detected a wave-V response with a median of 60 (55 to 75) dB nHL. This value was 20 dB higher than the preoperative BC thresholds, which were 40 (33.3 to 53.3) dB, and showed a moderate effect size (*R*^2^ = 0.32, *p* = 0.029). The coupling efficiency on the other hand (Vibrogam threshold—postoperative BC threshold) was −5 (−20 to 2) dB (Figure 3C) with a moderate to strong effect size (*R*^2^ = 0.37, *p* = 0.016). There was one outlier (patient no. 12, Table 1), where there was a marked ossification of the round window niche. A satisfactory intraoperative FMT coupling was not possible in this case, which was later confirmed by the postoperative coupling efficiency. ECochG measurement was not possible in this case due to poor SNR.

For ECochG measurements, the median signal threshold was 70 (55–77.5) dB nHL and lay 30 dB higher (Figure 3B) compared to the preoperative BC threshold (near-significant moderate to strong effect size, *R*^2^ = 0.48, *p* = 0.056). For ECochG (Figure 3D), the coupling efficiency showed no linear correlation (*R*^2^ = 0.02, *p* = 0.761).

### 3.3. Coupling modalities

**Figure 4:**
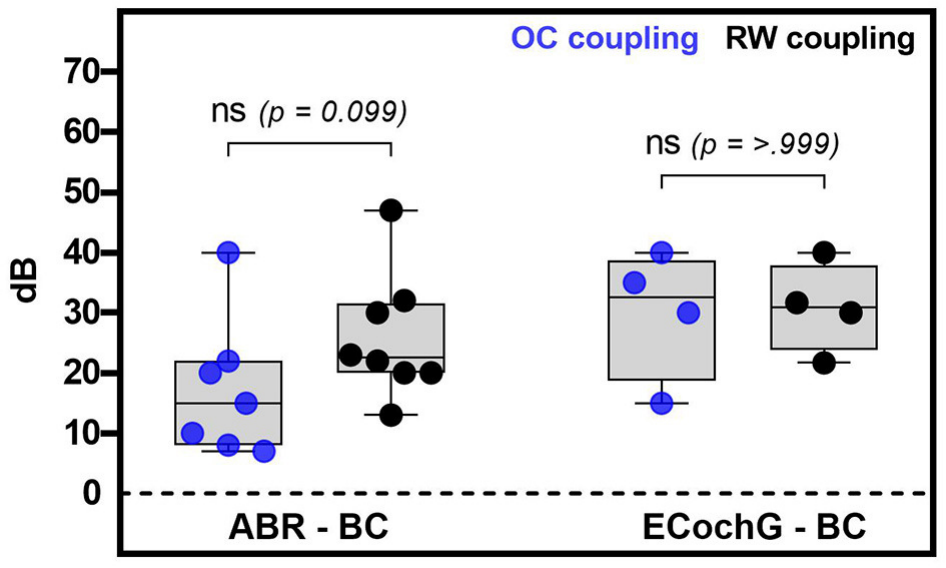
This figure shows the analysis of the coupling modality used in relation to the intraoperative electrophysiological (ABR or ECochG) minus the preoperative bone conduction thresholds. The blue dots in the graph represent coupling with the ossicular chain, while the black dots represent coupling with the round window. In this context, whiskers represent the minimum and maximum values, while boxes represent the median and quartiles. No significant difference was found between coupling with the ossicular chain and the round window for both methods.

Figure 4 displays the measured ABR and ECochG thresholds comparing the ossicular chain and round window coupling. The results of the rank test comparison indicated no statistically significant difference between the two coupling modalities for measuring with ABR (*p* = 0.099) or EcochG (*p* ≥ 0.999). However, looking at the ABR-BC thresholds, there was a trend toward better sound transmission when using a OC coupler. For both FMT placements, the ABR measurements lay 15 (8 to 22) dB (OC) and 22.5 (20 to 31.5) dB (RW) above the BC thresholds. For the EcochG recordings, these values were higher [32.5 (18.75 to 38.75) dB and 30.9 (23.8 to 37.9) dB, respectively], regardless of the coupling modality.

## 4. Discussion

The objective of this study was to compare the effectiveness of two electrophysiological methods, namely ECochG and ABR, in assessing the coupling efficiency during the implantation of AMEIs (Table 2). Our study yields three primary findings. First, intraoperative monitoring of coupling efficiency is feasible and can enhance the AMEI implantation procedure by enabling real-time feedback to the surgeon and a preliminary assessment of the patient's postoperative outcome. Second, we observed that ABR is a more sensitive method than ECochG for measuring coupling efficiency in middle ear implants, utilizing the same test setup, patient, and surgical environment. ABR also demonstrated higher feasibility and reliability in clinical application. Finally, we offer normative data for both techniques, which can aid other clinical centers in using intraoperative monitoring for AMEI placement.

**Table 2:** Comparative analysis of intraoperative monitoring (i.e., auditory brainstem response/ABR and electrocochleography/ ECochG).

***Auditory brainstem response (ABR)***	***Electrocochleography (ECochG)***
***Electrode location***	
(−) Measurement distant to cochlea	(+) Measurement close to cochlea
***Interferences***	
(+) Adhesive electrodes have higher noise rejection	(−) Manual electrode placement increases the susceptibility to noise interference
***Time of measurements***	
(+) Shorter, measurement independent of surgeon	(−) Longer, measurement dependent on surgeon (additional positioning of electrode, i.e., 45 s to 3 min)
***Coupling testing (reliability)***	
(+) Measurements are possible during FMT coupling, after coupling (intraoperatively until wound closure) and at any point postoperatively (longitudinal comparison)	(−) Measurements can only be performed intraoperatively as long as the promontory is accessible (no longitudinal comparison)
***Signal quality***	
(+) Lower risk of surface impedance changes on reference electrodes (−) Far field increases risk of electrophysiological side effects (e.g., muscle contraction).	(+) Detection in the near field may result in higher signal amplitudes
***Surgical handling***	
(+) No risk of affecting the coupling of the FMT (+) ABR measurements are independent form surgeon (no additional operative steps, good reproducibility, as the measurements are always performed in the same way)	(−) The placement of the measuring electrode may affect the coupling of the FMT (in case of physical contact) (−) Body liquids (e.g., blood trickling) has impact on impedance (−) Variation in positioning the measurements electrode has impact on the recordings

For the two techniques, the positive (+) and negative (−) aspects are shown.

### 4.1. Study cohort

In our cohort, we observed successful preservation of cochlear function in all 15 participants after AMEI implantation (as shown in Table 1). However, on average, there was a slight worsening of air conduction thresholds postoperatively, which was attributed to blind sack closure and reduced sound transmission. It is noteworthy that most study participants had a history of multiple ear surgeries or had been irradiated because of a malignancy. In these cases, efficient coupling of the FMT may be more difficult due to scar tissue formation. Furthermore, six study subjects had preoperative inner ear hearing thresholds near the implant's hearing indication range (≥ 60 dB HL). In such cases, even minor differences in coupling efficiency can have a significant impact on postoperative outcomes, highlighting the importance of intraoperative monitoring. Poor coupling can result in patient dissatisfaction and non-use of the implant.

### 4.2. ECochG

In our study, the process of obtaining threshold estimations using EcochG proved to be challenging. Only 8 out of 15 cases yielded successful measurements due to issues such as fluctuating impedance values. It is worth noting that previous studies have not reported the number of failed measurements, but rather only the successful ones (6–8). The existing literature on evaluating coupling efficiency in AMEIs has primarily focused on ABR measurements (9–12, 14–16). It should be noted that the ECochG measurement has limitations, as it requires active participation from the surgeon and is only feasible if the promontory is accessible during surgery. Furthermore, postoperative measurements cannot be conducted in the same manner, and alternative methods such as ECochG measurement via a tympanic electrode may not provide an identical test setup. These limitations should be taken into account when interpreting the results.

Our experience suggests that intraoperative ECochG measurements are highly dependent on the positioning of the measuring electrode. Despite our efforts to place the electrode as close as possible to the round window niche, the surgical approach and type of coupler used can limit this positioning. The transmastoid round window coupling technique can pose challenges in terms of electrode placement, as the position of the electrode must not interfere with the placement of the FMT.

Furthermore, we found that the average thresholds of the ECochG measurements were 30 dB higher than the preoperative BC threshold and thus higher than the ABR measurements. The correlation between ECochG values and preoperative BC thresholds was slightly better than ABR, but worse with vibrogram values. It is important to note, however, that caution should be exercised in interpreting these results, as the ECochG group in our study was relatively small.

### 4.3. ABR

We were able to successfully obtain ABR measurements from all participants in our cohort, both during and after surgery. In contrast to ECochG, we observed that ABR measurements could be more easily integrated into the surgical procedure, as they do not necessitate active intervention from the surgeon and are less prone to abrupt signal loss, such as that caused by impedance fluctuations.

In our study, the mean intraoperative ABR thresholds were found to be approximately 20 dB higher than the preoperative PTA of the bone conduction thresholds. Moreover, the coupling efficiency, which represents the difference between the vibrogram thresholds and postoperative bone conduction thresholds, showed a stronger correlation with the intraoperatively measured ABR thresholds compared to ECochG. Additionally, when comparing coupling efficiency values obtained intraoperatively and 4 weeks postoperatively, our results showed stable or slightly improved values with an average improvement of 5 dB in our study cohort. In terms of the various coupling modalities, there was a non-significant trend toward better outcomes with OC couplers, which is not surprising.

Comparison with previously published thresholds remains difficult due to the lack of consensus on measurement setup, stimulation type, and analysis methods. Geiger et al. (11) investigated the implantation of a Vibrant Soundbridge in 30 patients and reported that intraoperative thresholds were approximately 4 dB lower than the preoperative bone conduction threshold (median, pure tone mean of 0.5, 1, 2, and 4 kHz), which is in contrast to our results (Figure 3A, 20 dB). In a subsequent study, the same research group performed intraoperative monitoring in 14 revision cases and observed no significant correlation between preoperative bone conduction thresholds and intraoperative measurements (10). However, they did find a significant correlation between intraoperative measurements and postoperative vibrogram thresholds. It is challenging to draw direct comparisons between our results and theirs as they employed a different stimulus for intraoperative assessment and a prefitted audio processor.

Fröhlich et al. conducted a study of 18 patients with similar demographic and audiological characteristics to our cohort (17). They investigated the frequency-specific coupling efficiency and found a range of postoperative coupling efficiency from approx. −10 to 40 dB and coupling efficiency ranging from −13.30 to 41.7 dB (Figure 3C), which is comparable to our findings. It should be noted, however, that direct comparisons between our study and that of Fröhlich et al. should be made with caution because Fröhlich et al. used a preprogrammed sound processor with an attached insert earphone for each implantation, resulting in different intraoperative ABR measurements. A recent study conducted by Sprinzl et al. presented findings similar to our study in terms of test design, measurements, cohort, and data analysis with 14 AMEI implantations (16). Our results showed an intraoperative ABR threshold almost identical to theirs. In the comparison between both studies, a discrepancy of 13 dB was observed specifically for the intraoperative threshold when compared to the preoperative bone conduction (BC) threshold. It is important to note that when making comparisons between studies, differences in the signal analysis methods employed and variations in individual hearing thresholds must be considered, even if the study design is similar.

In conclusion, our results suggest that electrophysiological measurement of coupling efficiency is useful when placing the FMT in AMEIs. This is particularly important for round window coupling, which increases the degrees of freedom of possible FMT placements. In comparing the two measurement methods (ABR and ECochG), we used available hard- and software without the need for additional programming. Our measurement setup can therefore be replicated by other centers.

When comparing the two measurement methods, ABR measurements were significantly more practical, could be better integrated into the surgical procedure, were more robust and consistent, and were less susceptible to interference. Furthermore, the ECochG measurements can be conducted in the post-operative setting, enabling the assessment of FMT coupling over time and the longitudinal evaluation of its performance.

### 4.4. Limitations

A major limitation of our study is the lack of technical calibration of the audio processor for the frequency-specific properties of the FMT in stimulus and related coupling modality. Such an evaluation would be valuable in interpreting the results and selecting the optimal stimulus for threshold determination. Additionally, for the two electrophysiological measurements, we used two different stimuli. These stimuli were selected based on previous research where they were evaluated and proposed accordingly (8, 14). Caution should be exercised when making a direct comparison between the two results. Lastly, our study was limited by a small cohort size, and future research with larger sample sizes will be necessary to validate our threshold values.

## 5. Conclusion

Monitoring the coupling efficiency of AMEIs is crucial, particularly in patients with a round window coupler. In our comparative study between ABR and ECochG measurements, ABR performed significantly better in terms of its seamless integration into the surgical workflow, higher success rate of measurements, threshold distance to the effective hearing threshold, and the feasibility of postoperative measurements. These findings highlight the importance of selecting the appropriate measurement technique to ensure accurate and reliable monitoring of coupling efficiency in AMEIs.

## Data availability statement

The original contributions presented in the study are included in the article/supplementary material, further inquiries can be directed to the corresponding authors.

## Ethics statement

The studies involving humans were approved by Swiss Association of Research Ethics Committee (BASEC-ID no. 2019-00555). The studies were conducted in accordance with the local legislation and institutional requirements. The participants provided their written informed consent to participate in this study.

## Author contributions

TG, SW, and GM were involved in material preparation and data collection. Designed the study and data analysis were performed by TG and SW. TG performed the statistical analysis. MC, GM, LA, and SW provided resources and supervision. All authors contributed to this work and provided feedback on the final manuscript.

## References

[1] Ball GR Rose-Eichberger K. Design and development of the vibrant soundbridge–A 25-year perspective. J Hear Sci. (2021) 11:9–20. 10.17430/JHS.2021.11.1.1

[2] Labassi S Beliaeff M Péan V Van de Heyning P. The Vibrant Soundbridge^®^ middle ear implant: a historical overview. Cochlear Implants Int. (2017) 18:314–23. 10.1080/14670100.2017.1358913 28784040

[3] Pegan A Ries M Ajduk J Bedeković V Ivkić M Trotić R. Active middle ear vibrant soundbridge sound implant. Acta Clin Croat. (2019) 58:348–53. 10.20471/acc.2019.58.02.20 31819333 PMC6884373

[4] Ernst A Todt I Wagner J. Safety and effectiveness of the Vibrant Soundbridge in treating conductive and mixed hearing loss: a systematic review. Laryngoscope. (2016) 126:1451–7. 10.1002/lary.25670 26468033

[5] Sprinzl GM Schoerg P Muck S Jesenko M Speiser S Ploder M. Long-term stability and safety of the soundbridge coupled to the round window. Laryngoscope. (2021) 131:E1434–42. 10.1002/lary.29269 33210744 PMC8246711

[6] Colletti V Mandale M Colletti L. Electrocochleography in round window vibrant soundbridge implantation. Otolaryngol Head Neck Surg. (2012) 146:633–40. 10.1177/0194599811430808 22140205

[7] Mandalà M Colletti L Colletti V. Treatment of the atretic ear with round window vibrant soundbridge implantation in infants and children: electrocochleography and audiologic outcomes. Otol Neurotol. (2011) 32:1250–5. 10.1097/MAO.0b013e31822e9513 21897320

[8] Radeloff A Shehata-Dieler W Rak K Scherzed A Tolsdorff B Hagen R. Intraoperative monitoring of active middle ear implant function in patients with normal and pathologic middle ears. Otol Neurotol. (2011) 32:104–7. 10.1097/MAO.0b013e3181fcf167 20962700

[9] Fröhlich L Rahne T Plontke SK Oberhoffner T Dziemba O Gadyuchko M. Intraoperative recording of auditory brainstem responses for monitoring of floating mass transducer coupling efficacy during revision surgery-proof of concept. Otol Neurotol. (2020) 41:e168–71. 10.1097/MAO.0000000000002511 31663998

[10] Rak K Köstler C Geiger U Kaulitz S Herrmann D Shehata-Dieler W. Application of an intraoperative auditory brainstem response measurement system in active middle ear implant revision surgery. Otol Neurotol. (2023) 44:483–92. 10.1097/MAO.0000000000003851 37026817

[11] Geiger U Radeloff A Hagen R Cebulla M. Intraoperative estimation of the coupling efficiency and clinical outcomes of the vibrant soundbridge active middle ear implant using auditory brainstem response measurements. Am J Audiol. (2019) 28:553–9. 10.1044/2019_AJA-18-0066 31318578

[12] Verhaegen VJO Mulder JJS Noten JFP Luijten BMA Cremers CWRJ Snik AFM. Intraoperative auditory steady state response measurements during Vibrant Soundbridge middle ear implantation in patients with mixed hearing loss: preliminary results. Otol Neurotol. (2010) 31:1365–8. 10.1097/MAO.0b013e3181f0c612 20729778

[13] Cargnelutti M Cóser PL Biaggio EPV. LS CE-Chirp(^®^) vs. click in the neuroaudiological diagnosis by ABR. Braz J Otorhinolaryngol. (2016) 83:313–7. 10.1016/j.bjorl.2016.04.018 27297956 PMC9444743

[14] Cebulla M Geiger U Hagen R Radeloff A. Device optimised chirp stimulus for ABR measurements with an active middle ear implant. Int J Audiol. (2017) 56:607–11. 10.1080/14992027.2017.1314558 28420277

[15] Cebulla M Herrmann DP Hagen R Rak K. Intraoperative auditory brainstem response measurements via the vibrant soundbridge active middle ear implant: comparison of two methods. Am J Audiol. (2022) 31:261–7. 10.1044/20227_AJA-21-00208 35472299

[16] Sprinzl GM Schorg P Edlinger S Ploder M Magele A. Clinical feasibility of a novel test setup for objective measurements using the vibrant soundbridge. Laryngosc Investig Otolaryngol. (2022) 7:1113–9. 10.1002/lio2.839 36000035 PMC9392414

[17] Fröhlich L Rahne T Plontke SK Oberhoffner T Dahl R Mlynski R. Intraoperative quantification of floating mass transducer coupling quality in active middle ear implants: a multicenter study. Eur Arch Otorhinolaryngol. (2021) 278:2277–88. 10.1007/s00405-020-06313-z 32880736 PMC8165065

